# The Prince of Wales at Guy's Hospital

**Published:** 1923-08

**Authors:** 


### THE PRINCE OF WALES AT GUY'S HOSPITAL.

On Monday, July 2, the Prince of Wales visited Guy's Hospital, of which he is President, to open the new Anatomy, Biology and Physics Departments of the Medical School. The day was warm and sunny, and the gathering in the great open space known as the Park was gay with the academic robes of the men and the light dresses of the women. Not only were the windows of the Hospital crowded with nurses and patients, but the roofs of the rather squalid houses commanding even the most distant view of the proceedings were full of boys and girls anxious to catch a glimpse of the Prince. His Royal Highness, who passed to the platform amid terrific cheering, was accompanied by various officials of Guy's, and took his seat between Lord Goschen, the Treasurer of the Hospital, and Mr. Herbert Waring, Vice-Chancellor of the University of London. Lord Goschen then delivered a brief address, pointing out that the extension gives a completely new Anatomy Department, with ample accommodation both for teaching and research, not only in human anatomy but also in embryology and histology. The various laboratories are arranged in accordance with modern ideas, and they are well, though not lavishly, equipped. New Departments are also provided for the subjects of Biology and Physics, and the transference of the latter department from the main building to the extension sets free accommodation which enables the School to make further provision for organic and bio-chemistry, rendered necessary by the increasing importance of these subjects.

#### " A Great and Progressive Hospital."

The Prince in a happy little speech pointed out that Guy's had in the past been fortunate in its friends. " The new departments," he said, " are to a great extent a monument to their generosity and to their desire to benefit their fellow-men. As President of this Hospital I am only too glad to have this chance of thanking them whole-heartedly. But a great and progressive hospital like this, grateful though it be for past favours, must always have an anxious eye on the future. If its doors always stand open for those who need help, there is another door which must always stand just as wide for those who want to give help—the door of its treasury." The Prince then declared open the new buildings, which are the completion (at the cost of £100,000) of a scheme of reconstruction begun twenty-seven years ago, and unlocked the door of the extension with a golden key. He then inspected the buildings, and before his departure paid a very welcome visit to several wards.

